# Lanthanide Complexes in Recent *Molecules*

**DOI:** 10.3390/molecules27186019

**Published:** 2022-09-15

**Authors:** Takashiro Akitsu

**Affiliations:** Department of Chemistry, Faculty of Science, Tokyo University of Science, 1-3 Kagurazaka, Shinjuku-ku, Tokyo 162-8601, Japan; akitsu2@rs.tus.ac.jp; Tel.: +81-3-5228-8271

The MDPI journal *Molecules* publishes excellent papers on molecules in every issue, but I found that there are surprisingly only a few papers (3) and reviews (1) on rare-earth elements (lanthanide) in the category of inorganic chemistry these days. Let us analyze the point of focus by quoting the summaries of the four examples. It is not necessarily a high-impact trend, but it might be an important topic for *Molecules*. Somewhat classical analytical chemistry solution coordination chemistry, “complexation”, seems to be an old and new problem. In fact, there are many reports investigating the coordination of rare-earth ions by new organic chelate ligands.

Bellotti et al. presented a review article about “complexation” of not only lanthanide but also actinide ions by “Deferoxamine B” (O,O-bidendate chelate) reagent in solutions [1]. “Deferoxamine B is an outstanding molecule which has been widely studied in the past decade for its ability to bind iron and many other metal ions. The versatility of this metal chelator makes it suitable for a number of medicinal and analytical applications, from the well-known iron chelation therapy to the most recent use in sensor devices. The three bidentate hydroxamic functional groups of deferoxamine B are the centerpiece of its metal binding ability, which allows the formation of stable complexes with many transition, lanthanoid and actinoid metal ions. In addition to the ferric ion, in fact, more than 20 different metal complexes of deferoxamine b have been characterized in terms of their chemical speciation in solution.”

Avagyan and colleagues published two original studies about newly designed diamide compounds predominantly acting as a N,N-bidentate ligand (similar to 1,10-phenanthoroline potentially with O-sites of the amide) and possible “complexation” to lanthanide ions [2,3]. In such studies, they focused on the effects of substituent groups. “The first examples of 1,10-phenanthroline-2,9-diamides bearing CF_3_-groups on the side amide substituents were synthesized. Due to stereoisomerism and amide rotation, such complexes have complicated behavior in solutions. Using advanced NMR techniques and X-ray analysis, their structures were completely elucidated. The possibility of the formation of complex compounds with lanthanide nitrates was shown, and the constants of their stability are quantified.”; “An efficient approach to the synthesis of diamides of 4,7-difluoro-1,10-phenanthroline-2,9-dicarboxylic acid was elaborated. Direct nucleophilic substitution with 4,7-dichloro-1,10-phenanthroline precursors opened access to difluoro derivatives in high yield.”

Elenkova et al. reported Eu(III) complexes incorporating typical N,N-bidentate ligands mainly for the investigation of optical properties and coordination (developing luminescent materials) [4]. “New antenna ligand, 2-(phenylethynyl)-1,10-phenanthroline (PEP), and its luminescent Eu(III) complexes, Eu(PEP)_2_Cl_3_ and Eu(PEP)_2_(NO_3_)_3_, are synthesized and characterized. The synthetic procedure applied is based on reacting of europium salts with ligand in hot acetonitrile solutions in molar ratio 1 to 2. The structure of the complexes is refined by X-ray diffraction based on the single crystals obtained.”; “The newly designed complexes differ in counter ions in the inner coordination sphere, which allows exploring their influence on the stability, molecular and supramolecular structure, fluorescent properties and symmetry of the Eu(III) ion.”

About a decade ago, we published the preparation, crystal structure, magnetic and optical properties of Schiff base binuclear 3d-4f complexes in crystalline state, acetone solutions, and polymer matrix [5]. Contrary to 3d ions, which were kept by a N,N,O,O-tetradendate salen site, limited 4f ion (Gd(III)) surrounded by ten O atoms (O,O,O,O—tetradendate sites and three nitrates of bidendate chelates) could be possessed even in a solution. “Four chiral Schiff base binuclear 3d-4f complexes (NdNi, NdCu, GdNi, and GdCu) have been prepared and characterized by means of electronic and CD spectra, IR spectra, magnetic measurements, and X-ray crystallography (NdNi). A so-called artifact peak of solid-state CD spectra, which was characteristic of oriented molecules without free molecular rotation, appeared at about 470 nm. Magnetic data of the complexes in the solid state (powder) and in PMMA cast films or solutions indicated that only GdCu preserved molecular structures in various matrixes of soft maters.”

According to textbooks, lanthanide ions with large ionic radii are suitable for ligands with high coordination numbers and steric leeway. According to the HSAB principle, lanthanide ions prefer O atom coordination. Like other metal ions, lanthanide ions are said to contribute to stabilization by a chelating effect. At first glance, however, these represent a modest achievement among recent inorganic chemistry, but *Molecules*’ value as a medium for disseminating academic information lies in the fact that it publishes a variety of specific theories without omission as well as having an open access model.
